# In vitro evaluation of an intravenous microdialysis catheter for therapeutic drug monitoring of gentamicin and vancomycin

**DOI:** 10.1002/prp2.483

**Published:** 2019-06-26

**Authors:** Jackelien E. van der Mast, Maarten W. Nijsten, Jan‐Willem C. Alffenaar, Daan J. Touw, Wouter Bult

**Affiliations:** ^1^ Department of Clinical Pharmacy and Pharmacology University of Groningen University Medical Center Groningen Groningen The Netherlands; ^2^ Department of Clinical Pharmacy Máxima Medical Center Veldhoven The Netherlands; ^3^ Department of Critical Care University of Groningen University Medical Center Groningen Groningen The Netherlands; ^4^ Department of Pharmacy, section Pharmacokinetics, toxicology and targeting University of Groningen Groningen The Netherlands

**Keywords:** gentamicin, intensive care unit, intravenous microdialysis, therapeutic drug monitoring, unbound drug concentrations, vancomycin

## Abstract

A central venous catheter with a built‐in microdialysis membrane is available for continuous lactate and glucose monitoring in the intensive care unit (ICU). As this catheter might also be suitable for repeated measurements of unbound drug levels, we studied in vitro the feasibility of monitoring unbound antibiotic concentrations. The catheter was placed in various media at 37°C spiked with gentamicin or vancomycin. Dialysate fractions were repeatedly collected over 3 hours with a NaCl 0.9% perfusate flow of 5 μL/min. Total and unbound drug concentrations in medium and perfusate were measured by immunoassay. After 60 minutes stable recovery for both drugs was observed, with mean ±SD relative recoveries of vancomycin and gentamicin in human serum of 64% ±0.4% and 73% ±3%. The recoveries of the unbound concentrations were 91% ±3% and 91% ±4%. This intravenous microdialysis system may be a very useful platform for therapeutic drug monitoring in the ICU.

**Abbreviations**
CVC: Central venous catheters
ICU: intensive care unit
LLOQ: Lower Limit of Quantification
RRT: relative recovery of the total concentration
RRU: relative recovery of the unbound concentration
TDM: Therapeutic Drug Monitoring

## 1 INTRODUCTION

Patients in the intensive care unit (ICU) often require the concomitant administration of several drugs. However, ICU patients display marked differences in pharmacokinetics compared with healthy persons.^1^, ^2^ For example, changes in volume of distribution can result in inadequate plasma drug concentrations, whereas decreased clearance may increase the risk of toxicity.^2^, ^3^ Reduced protein binding resulting from a lower plasma albumin concentration may increase drug clearance thereby lowering the total drug concentration without effect on the unbound drug concentration with the risk of unnecessary increases of drug doses. Therapeutic drug monitoring (TDM) is therefore essential in these patients to optimize antimicrobial treatment.^4^ To adequately assess the patients’ pharmacokinetics multiple blood samples are required, which can be distressing to the patient. Moreover, the unbound drug concentration exerts the therapeutic effect and is the best predictor of the effectiveness and is highly variable in these patients,^5^ yet this requires a laborious and expensive separation step. A method for directly measuring the unbound concentration is preferred.^6^

**Figure 1:**
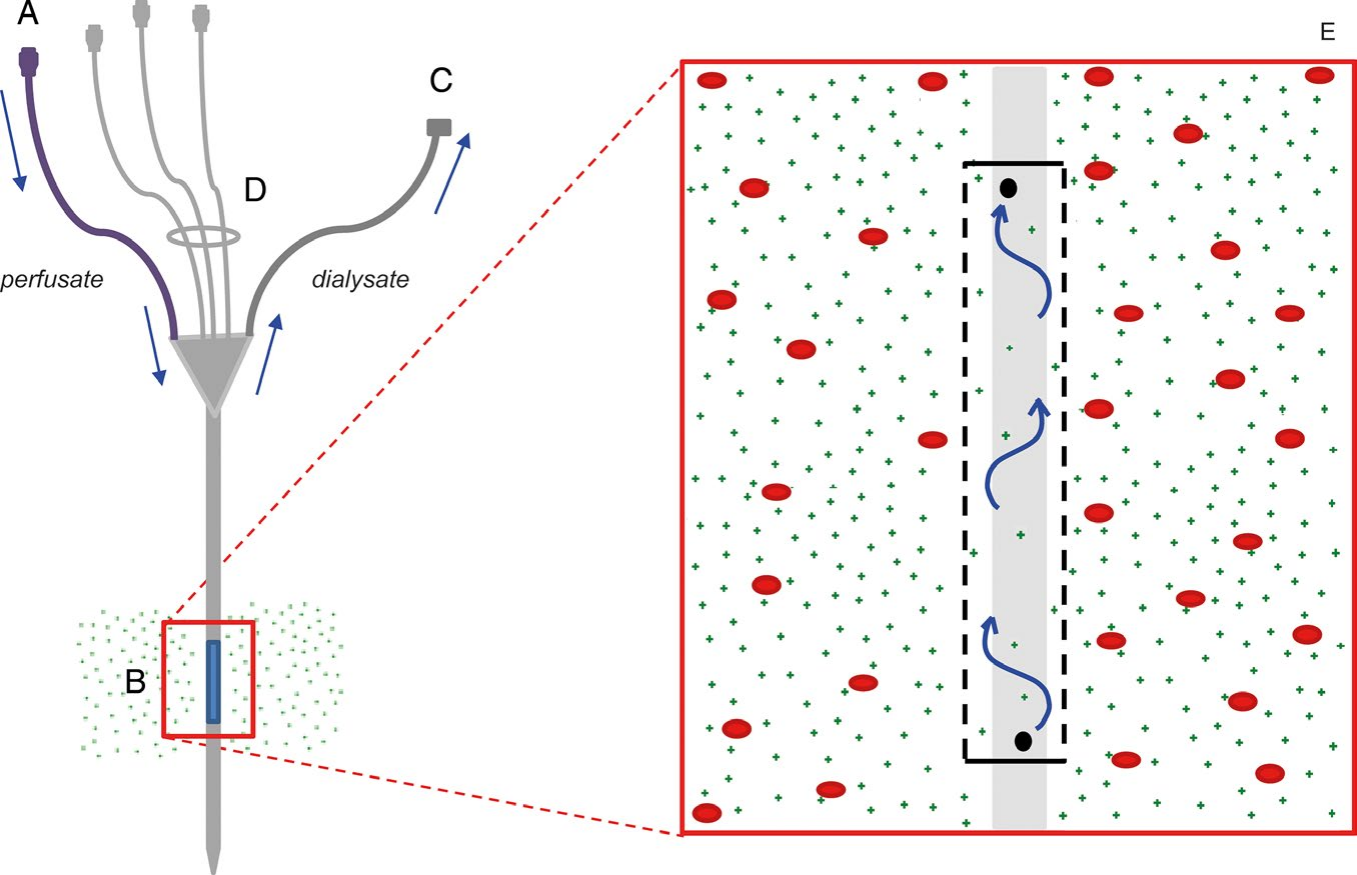
The EIRUS CVC: (A) perfusate inlet, (B) semipermeable membrane with a cutoff below 5000 Da, (C) microdialysate outlet, (D) three lumens for the administration of drugs, (E) an enlargement of the semipermeable membrane. Whereas large molecules (depicted as red dots) cannot pass the membrane, small molecules (<5000 Da, depicted as green dots) can pass it, thus arriving in the perfusate and creating a dialysate. The perfusion fluid flows from the perfusate inlet (A), via the distal end of the microdialysis chamber to the proximal site, and the dialysate leaves the catheter via the microdialysate outlet (C)

The commercially available intravenous microdialysis EIRUS^®^ catheter has been designed for use in ICU patients to continuously monitor lactate and glucose at the bedside.^7^ This catheter is a standard triple‐lumen intravenous catheter, which integrates a novel microdialysis function (Figure 1) which strongly reduces the need for lactate and glucose blood sampling for up to 30 days.^7^ Central venous catheters (CVC) are used in the majority of ICU patients.

We hypothesized that an intravenous microdialysis catheter may therefore serve as an ideal platform for repeated measurements of the circulating unbound concentrations of many drugs. The aim of this study was to investigate the usability of intravenous microdialysis for TDM of vancomycin and gentamicin in an in vitro setting.

## 2 MATERIALS AND METHODS

### 2.1 Materials

Vancomycin 1000 mg (Xellia Pharmaceuticals ApS, Copenhagen, Denmark) and gentamicin 10 mg/mL (Centrafarm B.V., Etten‐Leur, the Netherlands) were used as model drugs. Human serum was purchased from Millipore (Darmstadt, Germany), Human plasma (Omniplasma) and albumin (Albuman) were obtained from Sanquin, Amsterdam, the Netherlands. NaCl 0.9% was obtained from Baxter (Viaflo, Utrecht, the Netherlands). Ammonium acetate, disodium phosphate and potassium phosphate were used in the buffers and were obtained from Merck (Merck Millipore Ltd., Cork, Ireland). All other chemicals were of analytical grade and used as obtained.

### 2.2 Catheter

All experiments were carried out with EIRUS^®^ (Maquet Critical Care, Solna, Sweden) catheters 14G, triple lumen, 5000 Da cutoff. The microdialysis function was not affected when the catheter was used for infusions and drug administration, since the dialysis membrane is physically separated from and located “upstream” relative to the catheter tip (Figure 1). The microdialysis catheter uses a 0.9% NaCl carrier fluid as perfusate with a continuous flow of 5 μL/min.^8^

Figure 1 shows a schematic drawing of the catheter. The outer diameter of the CVC is approximately 2 mm. The microdialysis membrane is close to 3 mm in diameter and the effective length of the microdialysis chamber is approximately 35 mm, constituting a membrane contact surface of approximately 330 mm^2^.

Prior to use, the system requires equilibration for 10 minutes.^8^, ^9^ With a flow of 5 μL/min it takes 5 minutes between diffusion in the microdialysis chamber and collecting fractions of the dialysate at the microdialysis outlet (Figure 1C) resulting in a lag time of 5 minutes.^10^

CVC's were re‐used, cleaned between experiments with blank medium and perfusate. Prior to starting each experiment, a dialysate sample was measured to determine that the CVC's were free of drugs.

### 2.3 In vitro set‐up

Drugs were added to a test‐tube in a therapeutic relevant range, that is, vancomycin 15 to 22 mg/L and gentamicin 8 to 22 mg/L, containing a medium surrounding the catheter before the start of the microdialysis. The medium was constantly stirred to simulate blood flow and optimize microdialysis and kept at 37⁰C using a water bath. T = 0 was defined as the time the catheter was placed in the test medium. Samples were simultaneously collected from the medium and dialysate, to calculate the relative recovery (RR).

The perfusate inlet of the CVC was connected to an adjustable low‐flow pump (Alaris^®^ GH Syringe Pump, CareFusion, Rolle, Switzerland) at a flow rate that was used in previous studies 0.3 mL/h (5 μL/min).^7^, ^9^, ^10^, ^11^, ^12^, ^13^

### 2.4 Media

The in vitro testing started using a 0.9% NaCl solution as medium. In subsequent measurements, the medium complexity was gradually increased from saline to human serum:

Medium 1: NaCL 0.9% solution.

Medium 2: 0.2 M ammonium acetate buffer (set at pH 7.4 using ammonia).

Medium 3: 0.2 mmol/L phosphate buffer (pH 7.4).

Medium 4: 0.2 mmol/L phosphate buffer with albumin 40 g/L (pH 7.1).

Medium 5: 0.2 mmol/L phosphate buffer with albumin 40 g/L in NaCl 0.9% (pH 6.9).

Medium 6: 0.2 mmol/L phosphate buffer with albumin 40 g/L in NaCl 1.8%.

Human plasma.

Human serum.

### 2.5 Analysis of drug concentrations

Dialysate fractions of 50 μL (10 min) or 100 μL (20 min) and 100 μL samples of medium were simultaneously collected. The unbound fractions from the media were obtained using ultrafiltration (Centrifree^®^ Ultrafiltration Devices, Merck Millipore Ltd., Cork, Ireland) to separate the bound concentration and the unbound concentration. Gentamicin and vancomycin total concentrations and unbound concentrations were measured using an immunoassay (Abbott^®^ Architect c8000 analyzer, Abbott Laboratories, North Chicago, IL, USA), with a coefficient of variation < 5% and a Lower Limit of Quantification (LLOQ) of 0.5 mg/L and 2.00 mg/L, respectively. The intraday and interday precision and accuracy of this assay was determined by a recovery study on spiked samples. The accuracy of this assay is continuously monitored by participation in external quality control programs.^14^, ^15^

### 2.6 Determination of Relative Recovery (RRT and RRU)

The recovery was calculated using drug concentrations in the dialysate fractions and in the external medium. The recovery of the total concentration (RRT) is the ratio between the concentrations of the substance in the dialysate to the total concentration in the external media using the formula:

$$\mathrm{RRT}\left(\%\right)=\frac{\mathrm{concentration}\;\mathrm{substance}\;\mathrm{in}\;\mathrm{dialysate}\;\left(\mathrm{mg}/L\right)}{\;\mathrm{concentration}\;\mathrm{total}\;\mathrm{substance}\;\mathrm{in}\;\mathrm{external}\;\mathrm{medium}\;\left(\mathrm{mg}/L\right)}*100\%$$

The relative recovery of the unbound concentration (RRU) is the ratio between the concentrations of the substance in the dialysate to the unbound concentration in the external media using the equation:

$$\mathrm{RRU}\left(\%\right)=\frac{\mathrm{concentration}\;\mathrm{substance}\;\mathrm{in}\;\mathrm{dialysate}\;\left(\mathrm{mg}/L\right)}{\;\mathrm{concentration}\;\mathrm{unbound}\;\mathrm{substance}\;\mathrm{in}\;\mathrm{external}\;\mathrm{medium}\;\left(\mathrm{mg}/L\right)}*100\%$$

The mean RRT and RRU were calculated after 60 minutes.

### 2.7 Statistical analysis

All experiments were performed in triplicate. Mean and standard deviation were calculated. A t‐test was calculated using Excel (MS Office 2010).

## 3 RESULTS

Table 1 shows an overview of the results of the vancomycin measurements. As can be seen, the complexity of the medium affects the RRT. When albumin was added to the medium, RRT decreased. The mean RRT of vancomycin in human serum was 64% ±0.4% and 91% ±3% for the RRU.

**Table 1:** Experimental conditions and results for in vitro microdialysis

External medium Vancomycin	Total drug concentration medium (mg/L)	Unbound drug concentration medium (mg/L)	Concentration dialysate (mg/L)	RRT (%)	RRU (%)
Medium 1	17.1 (17.0‐17.3)	AI^a^	13.0 (12.1‐13.5)	76.3 ± 4.5	AI^a^
Medium 2	19.0 (18.6‐19.6)	AI^a^	14.6 (13.4‐15.3)	78.5 ± 1.8	AI^a^
Medium 3	17.5 (16.8‐18.1)	AI^a^	16.2 (16.1‐16.4)	92.9 ± 3.3	AI^a^
Medium 4	17.6 (17.5‐17.6)	6.7 (6.6‐7.4)	9.3 (8.8‐9.8)	53.1 ± 2.8	137.2 ± 7.8
Medium 5	17.3 (17.3‐17.4)	10.3 (10.3‐10.4)	12.0 (11.7‐12.3)	68.9 ± 1.6	116.7 ± 2.9
Medium 6*	18.9 (18.9‐18.9)	13.3 (13.3‐13.3)	14.0 (13.8‐14.2)	74.5 ± 1.7	105.4 ± 1.6
Human plasma	19.2 (15.5‐21.9)	13.2 (10.0‐14.9)	11.6 (10.0‐12.7)	59.9 ± 4.9	89.2 ± 4.9
Human serum	19.0 (18.4‐19.4)	13.2 (13.2‐13.2)	12.1 (11.6‐12.4)	63.6 ± 0.4	90.7 ± 2.9
Gentamicin					
Medium 3	21.8 (21.6‐21.9)	AI^a^	21.5 (21.1‐21.8)	98.7 ± 0.5	AI^a^
Medium 5	11.6 (11.5‐11.6)	7.3 (7.2‐7.4)	7.5 (7.4‐7.8)	65.2 ± 2.2	103.6 ± 1.1
Human plasma**	10.7 (8.9‐12.6)	8.9 (7.7‐10.9)	8.4 (7.1‐9.6)	78.9 ± 2.7	91.7 ± 2.9
Human serum*	9.8 (7.8‐11.8)	7.7 (6.6‐8.9)	7.1 (5.8‐8.3)	72.6 ± 3.1	90.7 ± 3.7

Medium and dialysate concentrations, relative recovery of the total concentration (RRT) and recovery of the unbound concentration (RRU) for vancomycin and gentamicin using different external media. Mean ±SD. The human serum experiment of gentamicin and medium 6 of vancomycin were repeated twice (*) and the human serum experiment four times (**).

a AI: assumed identical to the total concentration as all the antibiotic was assumed unbound in these media that contained no protein.

The results of the gentamicin measurements are also summarized in Table 1. The RRT of gentamicin in a phosphate buffer was close to 100%. When albumin was added to the phosphate buffer, RRT decreased to 65%. A RRU of 104% was observed. The mean RRT for gentamicin in human serum was 73% ±3.1% and 91% ±3.7% for the RRU.

**Figure 2:**
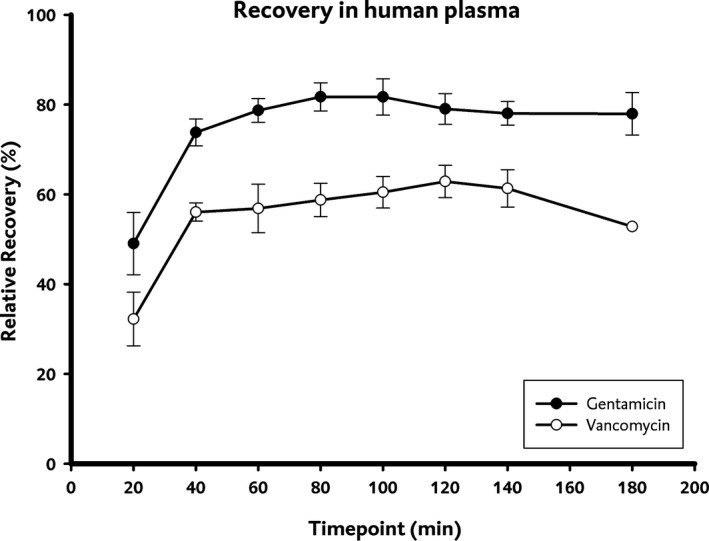
Relative recovery of the total concentration (RRT) of gentamicin and vancomycin in human plasma

Figure 2 shows the RRT of the total drug concentration of gentamicin and vancomycin in human serum, expressed in the duration of microdialysis. As can be seen in the Figure 2, after one hour equilibrium was achieved. The mean RRT of gentamicin in human plasma during equilibrium was 78.9%.

The stability of drug recovery was tested using a *t*‐test. For both gentamicin and vancomycin a constant recovery was observed after 60 minutes: the t‐test showed significant difference prior to 60 minutes, and no significant difference after 60 minutes.

## 4 DISCUSSION

In this study, we have evaluated the in vitro suitability of an intravenous microdialysis CVC for TDM of vancomycin and gentamicin. The EIRUS CVC is an attractive device for continuous lactate and glucose measurements in critically ill patients,^7^, ^8^, ^9^, ^10^, ^11^, ^12^, ^13^ due to a high surface area and a high perfusion flow rate compared to microdialysis in tissue or peripheral veins. In addition, combination of the microdialysis membrane on a CVC avoids the local trauma response and inflammatory response observed with tissue microdialysis.^16^ Moreover, tissue drug concentrations may not be the same as circulating concentrations or concentrations in the relevant tissues. Therefore, intravenous microdialysis is preferable.

The molecular weight membrane cut‐off (5000 Da for EIRUS) allows lactate (89 Da) and glucose (180 Da) to freely cross the membrane. Usually, molecules with a molecular weight of one‐fourth of the molecular weight cut‐off of the membrane can reach an acceptable recovery level.^16^ Gentamicin has a molecular weight of 478 Da and vancomycin has a molecular weight of 1486 Da. These molecular weights are approximately 2 fold and 8 fold higher than glucose.^17^ Moreover, vancomycin was selected as a “worst case” scenario drug. If vancomycin with its relatively large molecular weight and positive charge at physiological pH would pass the membrane we hypothesize that many smaller and less charged drugs of interest will pass the membrane.

Recovery is influenced by the membrane area: the larger the surface area, the higher the recovery since equilibrium is reached faster.^16^ The intravenous microdialysis catheter had a membrane surface area of approximately 330 mm^2^: two orders of magnitude larger than most subcutaneous microdialysis probes, resulting in a faster equilibrium and making it ideal for TDM. Another advantage is that samples are free of enzymes after dialysis, which results in more stable samples that require less processing.^16^

The composition of the dialysate and the constitution of the sample matrix influences the recovery.^16^ When increasing the complexity of the sample matrix the RRT decreased due to protein binding of the drug. The perfusion fluid was not varied and the standard EIRUS perfusate was used: NaCl 0.9%.^9^ The time required to achieve a stable RRT or RRU was approximately 1 hour (P < 0.05), which is similar to the lactate and glucose measurements.^9^ This lag time is likely due to the time needed to flush the system to stabilize non selective adhesion in the microdialysis membrane and catheter affecting the recovery.

When increasing the osmolarity from 308 to 616 mOsm/L, the recovery of vancomycin increased from 69% to 74% likely caused by the concentration gradient and increased diffusion rate. Differences in osmotic pressure result in a convective flow and is induced by the movement of water molecules across the semi‐permeable membrane which in turn affects the rate of passage of small water‐soluble molecules.^18^

The results of this study show a steady recovery of both drugs in the dialysate from buffer, human serum and human plasma. Intravenous microdialysis may therefore provide reliable information to improve TDM in the critically ill: measurements can be performed continuously, allowing for immediate action once concentration deviates from the desired value. In addition, only the unbound drug can pass the membrane: the unbound drug is the active drug concentration which can fluctuate in critically ill due to variation of the protein concentration.^6^ Reported unbound fractions for vancomycin vary between 45% and 95%.^19^, ^20^ In addition, due to large fluctuations in the unbound concentration of vancomycin in critically ill patients, measuring the unbound concentration might further improve TDM of vancomycin.^5^ The use of a combined CVC with an integrated microdialysis system, such as the EIRUS, is a potential approach for TDM of unbound drug concentrations in the ICU. Further research could focus on trough and peak sampling and prolonged measurements. Also studies on drugs with higher protein binding where measurement of the unbound concentration is particularly relevant (eg, ceftriaxone) are warranted to further investigate the opportunities and limitations of intravascular microdialysis. Although we restricted ourselves to the fluid and flow conditions designed for glucose and lactate measurements, other perfusates or flows might allow even better drug recovery.

In conclusion, this study shows that intravenous microdialysis is an accurate and useful platform for unbound drug monitoring of gentamicin and vancomycin without serial blood sampling and could be very useful in the ICU. This method may improve patient care as well comfort and deserves further clinical evaluation.

## DISCLOSURE

The authors declare that they have no competing interests.
